# Evaluation of a pressure sensitive walkway for objective gait analysis in normal and arthritic domestic ducks (*Cairina moschata domestica*)

**DOI:** 10.1371/journal.pone.0220468

**Published:** 2019-07-25

**Authors:** Julie D. Sheldon, Michael J. Adkesson, Matthew C. Allender, Ryan S. Bailey, Jennifer N. Langan, Sathya K. Chinnadurai

**Affiliations:** 1 Chicago Zoological Society, Brookfield Zoo, Brookfield, Illinois, United States of America; 2 Illinois Zoological and Aquatic Animal Residency Program, Urbana, Illinois, United States of America; 3 Wildlife Epidemiology Laboratory, Department of Veterinary Clinical Medicine, College of Veterinary Medicine, Urbana, Illinois, United States of America; 4 Premier Veterinary Group, Chicago, Illinois, United States of America; 5 University of Illinois, College of Veterinary Medicine, Urbana, Illinois, United States of Ameirca; Tokat Gaziosmanpasa University, TURKEY

## Abstract

Objective gait evaluation with a pressure sensitive walkway (PSW) has been used to assess welfare of poultry and to assess lameness and response to therapy in domestic mammals. Objective gait analysis of birds with lameness due to pododermatitis, osteoarthritis, and other common diseases could provide non-biased assessment and therapeutic monitoring for zoo clinicians. The objective of this study was to evaluate the use of a PSW for objective gait analysis in normal domestic ducks (*Cairina moschata domestica*) and those with experimentally induced arthritis. Eighteen healthy adult ducks walked across the PSW four times in each experiment at each time point. For experiment 1, gait parameters (step and stride distances and velocities, maximum force, impulse, and peak pressure) were calculated for each foot in each duck (time 0). For experiment 2, six of these ducks were randomly selected, anesthetized, and administered a unilateral intra-tarsal injection of monosodium urate solution to induce arthritis. Serial PSW trials were repeated at 1, 2, 3, 4, 8, and 24 hours post-injection. Gait parameters were calculated and compared at each time point, including baseline at time 0. Among the normal ducks, there were no significant differences between right and left feet for any gait parameter. Maximum force and impulse were significantly lower for the affected limb at the 3- and 4-hour time points in ducks with unilateral induced arthritis. This asymmetry was resolved by 8 hours post injection. This PSW transient arthritis model allows for objective assessment of lameness in domestic ducks with maximum force and impulse serving as the most sensitive gait parameters for lameness detection. This method has potential as a model to assess analgesic efficacy for zoo-housed waterfowl and other avian species.

## Introduction

Lameness due to pododermatitis, osteoarthritis, and nutritional deficiencies are common health conditions in birds across taxa with significant impacts on welfare in human care settings. Objective gait evaluation with a pressure sensitive walkway (PSW) has been used as a tool for welfare assessment in poultry, as well as assessment of lameness and response to therapy in domestic mammals [1–5]. Objective gait analysis of birds with lameness-causing diseases could provide non-biased assessment and therapeutic monitoring for zoo clinicians.

Avian analgesic efficacy studies use validated nociception models to establish evidence-based clinical standards for appropriate medications and dosing regimens, improving health and welfare of avian species [6–12]. The thermal threshold model conditions a bird to stand on a perch that differentially increases in temperature under one foot causing the bird to lift its foot to escape the stimulus. With effective analgesic treatment, the bird will require a stronger stimulus to lift its foot (increased thermal threshold) [6,8–10]. Monosodium urate (MSU) induced arthritis transiently mimics articular gout [13,14]. An incapacitance meter perch measures the amount of weight placed on a limb that has had a MSU solution injected into the tarsal joint. With effective analgesia, the bird will bear more weight on the affected limb than without treatment [7,11,12]. The majority of efficacy studies have been performed in psittaciformes and falconiformes, measuring weight-bearing at a standstill. There has yet to be a dynamic avian nociception model developed to evaluate lameness and response to treatment in aquatic birds at a walk or run.

Traditional gait assessment in veterinary medicine is based on skilled subjective evaluation, but remains flawed due to “caregiver” placebo bias, variability between observers, and differences in evaluation based on variations in gate dynamics between species (e.g. waddling penguin, walking ratite, hopping passerine) [15–17]. Objective gait analysis methods commonly used in veterinary medicine, such as force plate (in-ground plate that measures three-dimensional forces of a single step) and kinematic analysis (measurement of angles and motions of anatomic structures via camera recordings), have been validated primarily for canine and equine patients [15]. These methods, while useful, can be challenging to adapt to non-domestic species due to portability, cost, inflexibility related to patient size and gait type, and behavioral compliance with evaluation methods [15].

The pressure sensitive walkway (PSW) is a different method of objective gait analysis that provides temporospatial information using a pressure-sensing mat and communicates with a computer software system to calculate several gait parameters. The mat contains a matrix of resistive thin force sensors that gather information on the vertical ground reaction forces and contact area of the weight-bearing foot as the person or animal walks across the mat [18,19]. Advantages of the PSW include portability, ability to measure multiple steps in a single trial (as opposed to static step analysis with the force plate), ability to easily evaluate gait symmetry, and flexibility in patient size. Objective gait assessment technology has been used in human, domestic animal, production animal (poultry, bovine, and porcine) and has identified some differences in gait parameters that subjective evaluation has missed [1,2,4,20–27]. The PSW has yet to be utilized for zoological species in human care or as an avian analgesia efficacy tool using MSU-induced arthritis or lameness case-control studies.

This study evaluated the PSW as an objective gait analysis tool for normal ducks and those with experimentally induced arthritis as an avian model with the goal of using it for future analgesia efficacy studies. Authors hypothesize the PSW will successfully measure objective gait parameters of ducks and that the values will not differ significantly between right and left feet. In addition, authors predict a unilateral tarsal MSU injection will cause transient lameness in domestic ducks that will be detectable and quantifiable by the PSW.

## Materials and methods

This study was approved by the University of Illinois Institutional Animal Care and Use Committee (protocol # 17226). Twenty adult Muscovy ducks (*Cairina moschata domestica*) of unknown sex were acquired from a university-approved vendor. Ten ducks were housed in two rooms (9m x 4m) each containing a longitudinal pool (7.6m long x 1.4m wide, 9-23cm deep). Floors were sealed, smooth, painted concrete. One side of the room was filled with continuously flowing water and a deep end for swimming. The other side had varied substrate of rubber coated platforms, outdoor carpet, and pine shavings that were replaced daily. Water and poultry feed (Purina Premium Poultry Feed, Flock Raiser Crumbles, Purina Animal Nutrition LLC, Shoreview, Minnesota 55126, USA) were provided ad libitum. The rooms were pressure-washed and cleaned at least once daily.

Five days after arrival, each duck was weighed and had a complete physical examination performed by a veterinarian (JDS). Animals were individually identified using 1–2 plastic colored cable ties placed around either the right or left tarsometatarsus. Eighteen ducks met the inclusion criteria of lacking foot lesions or apparent lameness. Ducks were allowed to acclimate to their environment for three weeks. Health was assessed through the duration of this study via daily visuals by animal care staff and physical examination including body weight at intake and before each experiment.

**Fig 1:**
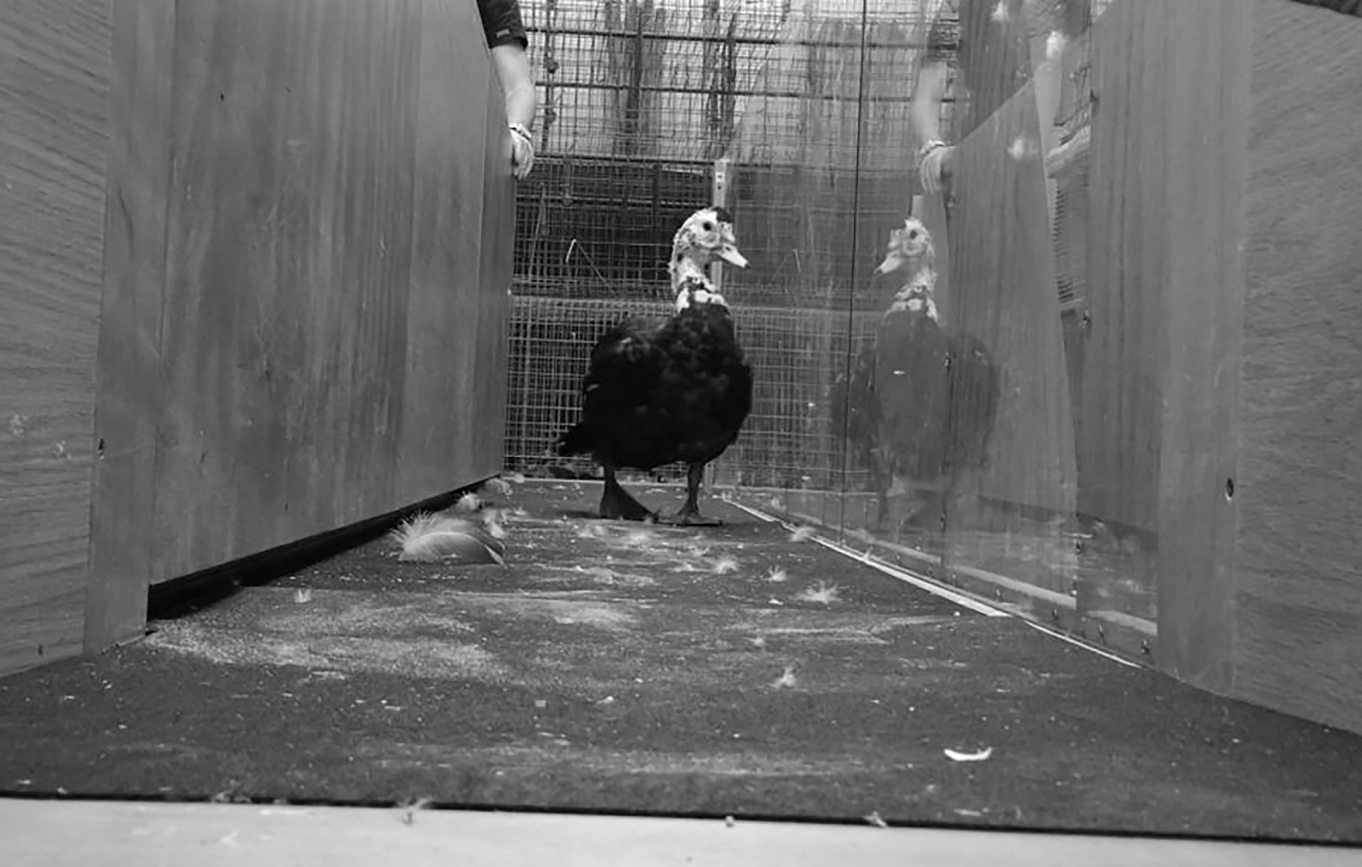
Pressure sensitive walkway. Muscovy duck (*Cairina moschata domestica)* on pressure sensitive walkway (PSW) within a custom-designed chute composed of wooden and acrylic panels.

For experiment 1, baseline objective gait analysis was performed. A 3-meter long pressure sensitive walkway (PSW; Tekscan Walkway 7 System; Tekscan, South Boston, Massachusetts 02127, USA) covered with a 2mm thickness smooth rubber mat (Multy Home LP, Concord, Ontario, Canada) was set up in an adjacent room. Custom-designed panels were added in order to help guide the ducks down the walkway (Fig 1). One side was wooden and the other was clear acrylic for observational purposes. The PSW was calibrated for an appropriate body weight range for ducks. Each duck was encouraged to walk down the PSW at least 4 times (4 trials, or repeats) by placing an individual on the ground at one end of the PSW and allowing it to naturally walk away from the person toward the other end of the PSW. If a full trial, defined as uninterrupted complete step readings for at least half the length of the PSW, was not obtained, the trial was repeated until 4 full trials were collected for each animal. Exclusions included partial foot strikes on the edges of the PSW, changing direction of movement, stopping, or partially taking flight. The walkway software measured and calculated several temporospatial parameters for each animal and each limb. Parameters included number of strikes; cadence (steps/min); gait time (sec), distance (cm) and velocity (cm/sec); stance and swing time; step time, length, width, and velocity; stride time, length and velocity; maximum force in kilograms and percent of body weight; impulse in kilogram meter per second and percent of body weight meter per second; and maximum peak pressure. Gait is defined as one full cycle of the same event (i.e. left foot touching the ground) and is made up of the stance phase (foot in contact with ground) and swing phase (foot not in contact with ground). A stride is defined as the distance that the right and left foot moves forward during the gait cycle while a step is the distance that only the right or left foot moves forward. Step width is the lateral distance between the right and left foot strike [5,28].

For experiment 2, six ducks were randomly selected for the MSU lameness induction model using a computer based random number generator. This sample size was chosen because the remaining 12 ducks were used for a larger study [29]. MSU crystal solution (3%) was prepared in accordance with methods described previously [11,12]. Specifically, 5mL 1N NaOH was added to 195mL sterile water and pH was adjusted to 12. The alkaline solution was boiled and stirred, 1g of uric acid was added, boiled for another 5 minutes, and then was removed from heat. An additional 1N NaOH was added until the solution cleared. This sat at 23°C for 48 hours, pH was adjusted to 8 using NaOH, and sat at 23°C for 48 more hours. It was centrifuged at 693xg for 15 min, the supernatant was decanted, and the crystals were washed in sterile saline (250mL). The solution was centrifuged again at 693xg for 20 min, and the supernatant decanted. Crystals were re-suspended in 25mL sterile saline in a sterile glass flask which was then capped and autoclaved at 93.3°C for 2 hrs. The suspension was pipetted with continuous stirring into sterile 30ml glass vials, centrifuged at 390xg for 15 min, and supernatant decanted. Then 33mL of sterile saline was used to suspend the crystal and make a 3% MSU solution. The presence of crystals in solution was confirmed by microscopic examination.

**Fig 2:**
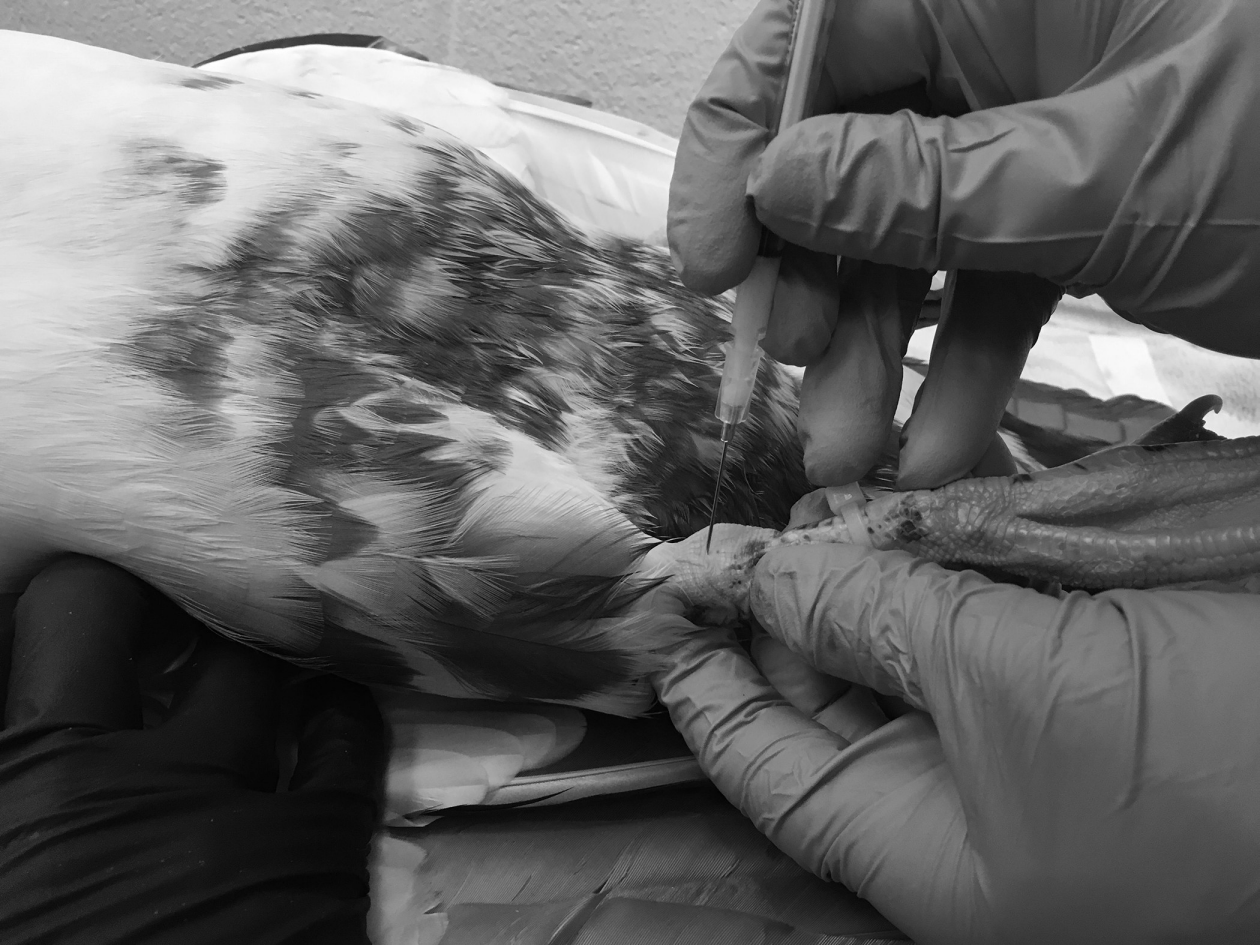
Arthritis induction. Intratarsal injection of monosodium urate solution in an anesthetized Muscovy duck (*Cairina moschata domestica)*.

Baseline objective gait analysis (time 0) was performed using the same methodology as experiment 1 immediately before the lameness induction procedure. For each of the six ducks, general anesthesia was induced using a non-rebreathing circuit with 5% isoflurane and 100% oxygen at 1L/min via facemask under manual restraint. Delivered isoflurane percentage was adjusted to maintain a moderate to light plane of anesthesia as measured by examining muscle tone, palpebral reflex, heart rate, and respiratory rate. Continuous monitoring of heart rate and rhythm using a stethoscope, and respiratory rate by visual observation were performed. Once at a working plane of anesthesia, the tarsus was aseptically prepared, the MSU solution was thoroughly mixed, and 0.2mL was injected into the right or left (side determined by a coin-toss) tarsal joint using a 1-cc syringe and 22-ga, 1-in hypodermic needle (Fig 2). Correct placement was confirmed via aspiration of synovial fluid prior to injection and palpation of joint capsule expansion following injection. Following injection isoflurane was immediately discontinued and ducks were recovered from anesthesia under manual restraint, followed by undisturbed time in a holding kennel for 30–60 min.

Following recovery, serial PSW timepoints using the same methodology as experiment 1 were performed at 1, 2, 3, 4, 8, and 24 hours post MSU injection. Ducks were held in an individual plastic or metal kennel between the 1, 2, 3, and 4 hour time points. They were placed in a larger kennel with food and water between the 4 and 8 hour time points and then returned to their normal housing until the 24 hour time point the following day. After the 24 hour PSW time point, each duck was anesthetized with alfaxalone (5–10 mg/kg intramuscular or intravenous; Alfaxan; alfaxalone, 10mg/ml, Jurox Pty Limited, Rutherford, NSW 2320, Australia) and euthanized with an overdose of intravenous pentobarbital (Euthasol; pentobarbital sodium 400mg/ml, Le Vet. Pharma BV, Wilgenweg 7, 3421 TV, Oudewater, The Netherlands) based on IACUC protocol requirements. Carcasses were incised along the coelom, sex was determined, and then were appropriately disposed of. Six of the remaining ducks from experiment 1 were used in a pharmacokinetic study prior to the current study, and the remainder of the ducks were used in a pharmacodynamics study after the current study [29,30].

For statistical analysis of experiment 1, the average of the four trials, or technical repeats, for each parameter and each foot were calculated. To assess repeatability of the 4 PSW trials, the within-case coefficient of variance (wCV = standard deviation/mean) was performed for the 4 trials of each gait parameter for each duck [31]. Descriptive statistics and absolute value of the difference between right and left limb (|R-L|) were calculated for each gait parameter for all ducks. Based on previous PSW gait analysis studies across species, asymmetry between limbs is a consistent method for gait abnormality identification [3,4,15,23,25,26]. Furthermore, previous MSU arthritis induction studies used asymmetry of weight bearing in Amazon parrots to quantify effectiveness of induction and treatment [11,12]. Normality was tested using the Shapiro-Wilk test. Values between R and L feet were compared using a paired t-test or Wilcoxon signed rank test for normally and non-normally distributed data, respectively. For experiment 2 statistical analysis, the average of the four trials for each parameter was calculated. A repeated measures analysis of variance was performed for each parameter and absolute value of the difference between right and left limb (|R-L|) at time points 0–24 hrs for all six ducks. Statistical significance was determined at a level of p < 0.05. All statistical analyses were performed using commercial software (Microsoft Excel for Mac 2011; Microsoft, Redmond, Washington 98052, USA; SPSS Version 24; IBM Statistics, Chicago, IL 60606, USA).

## Results

All eighteen ducks were in good body condition and had no significant abnormalities upon initial physical examination. The ducks remained healthy throughout the study period (all maintained or gained weight and none had abnormalities on physical examination prior to each experiment) and successfully completed the PSW trials. Of the 72 trials analyzed in experiment 1, 10 were repeated once and 5 were repeated twice in order to obtain a complete set of 4 adequate trials. The mean ± standard deviation (range) wCV for all gait parameters was 0.23 ± 0.09 (0.13–0.46) which is slightly higher than what is considered ideal for biological markers (0.1–0.2) [31]. Descriptive statistics for body weight and gait parameters, in addition to wCVs, for experiment 1 are provided (Tables 1 and 2). Among the ducks in experiment 1, there were no significant differences between right and left feet for any gait parameter.

**Table 1:** Experiment 1: Normal duck gait analysis.

**Body weight (kg)**	2.25 (1.74–3.65)
**Cadence (steps/min)**	342.0 ± 62.7 (314.0–370.0); 0.17
**Gait distance (cm)**	222.0 ± 42.0 (203.0–241.0); 0.27
**Gait time (sec)**	1.56 (1.13–3.72); 0.42
**Gait velocity (cm/sec)**	147 ± 39.1 (129–165); 0.26

Objective gait analysis of normal ducks (*Cairina moschata domestica*; n = 18) as measured by a pressure sensitive walkway. Values reported as mean ± standard deviation (95% confidence interval); mean wCV, or median (range); mean wCV if data not normally distributed (Shapiro-Wilk Test p < 0.05).

**Table 2:** Experiment 1: Normal ducks gait analysis continued.

Parameter	Left	Right	|Right–Left|	wCV
Stride length (cm)	51.3 ± 7.1 (48.1–54.5)	50.5 ± 7.5 (47.2–53.9)	1 (0.1–9.3)	0.17
Stride time (sec)	0.36 (0.25–0.67)	0.37 ± 0.08 (0.34–0.4)	0.01 (0–0.08)	0.19
Stride velocity (cm/sec)	148.0 ± 37.7 (131.0–165.0)	147.0 ± 37.6 (130.0–164.0)	2.7 (0.4–10.4)	0.26
Step length (cm)	26.2 ± 5.0 (23.9–28.5)	26.1 ± 4.7 (24.0–28.2)	1.4 (0.2–15.0)	0.21
Step time (sec)	0.19 ± 0.04 (0.17–0.21)	0.19 (0.12–0.34)	0.02 (0–0.09)	0.21
Step velocity (cm/sec)	146.0 ± 41.0 (128.0–164.0)	148.0 ± 37.6 (131.0–165.0)	9.2 ± 5.8 (6.6–11.8)	0.27
Step width (cm)	8.5 ± 1.4 (7.8–9.1)	8.5 ± 1.4 (7.7–9.2)	0.5 (0–1.2)	0.15
Stance time (sec)	0.17 (0.11–0.84)	0.19 ± 0.06 (0.16–0.22)	0.01 (0–0.62)	0.25
Swing time (sec)	0.19 (0.13–0.33)	0.19 ± 0.03 (0.18–0.20)	0.01 (0–0.24)	0.46
Max force (% BW)	120.3 ± 15.0 (113.6–127.1)	122.3 ±12.5 (116.7–127.9)	4.0 (0.1–19.1)	0.13
Impulse (%BW*sec)	13.5 (9.4–36.7)	13.2 (9.1–25.1)	0.5 (0–20.9)	0.18
Max Peak Pressure (kPa)	73 ±15 (66–80)	76 ± 14 (70–82)	8± 5 (6–11)	0.18

Objective gait analysis of normal ducks (*Cairina moschata domestica*; n = 18) as measured by a pressure sensitive walkway. Values reported as mean ± standard deviation (95% confidence interval) or median (range) if data not normally distributed (Shapiro-Wilk Test p < 0.05). wCV = within-case coefficient of variance for each gait parameter.

**Fig 3:**
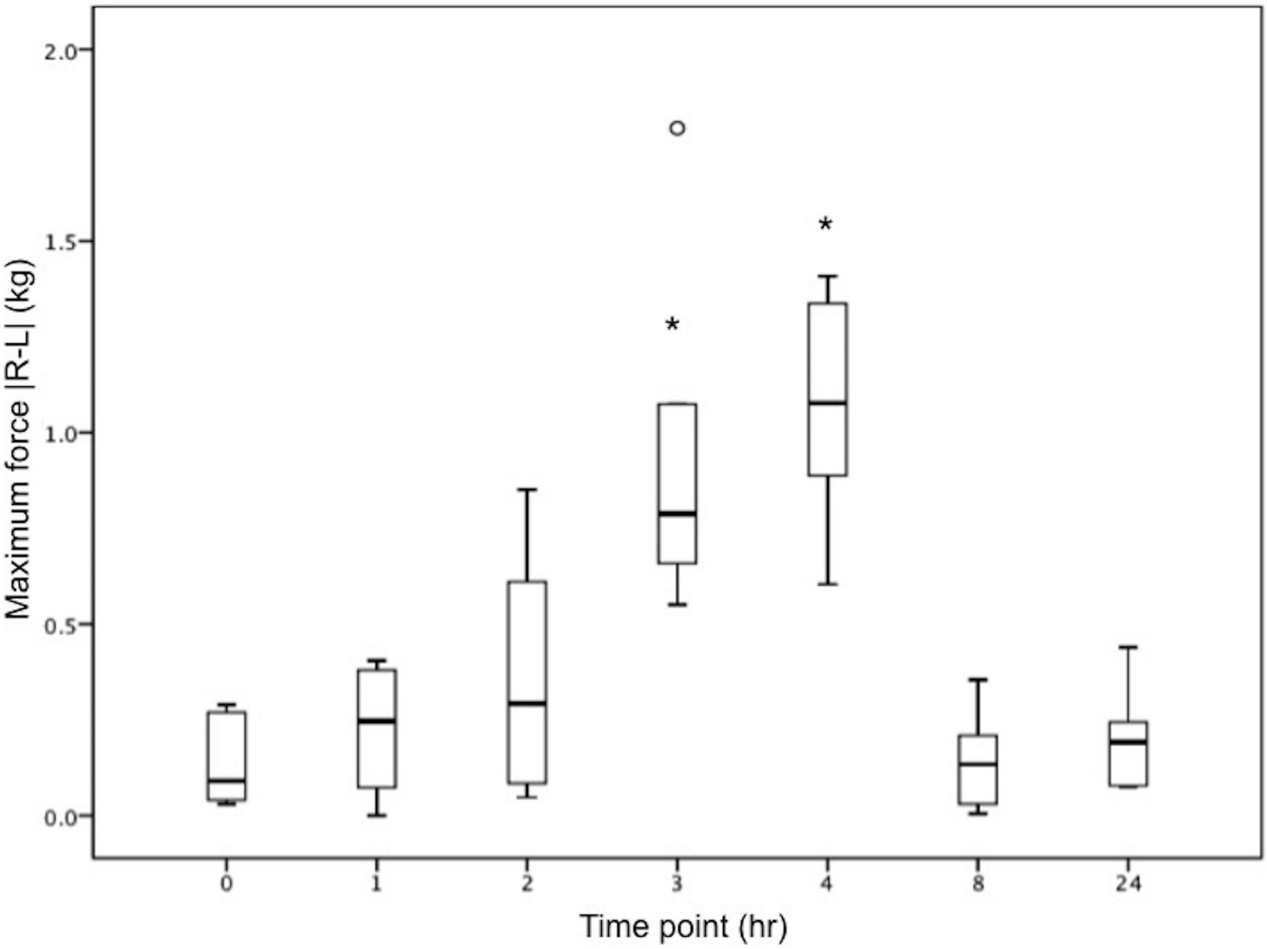
Maximum force. Boxplot depicting the absolute value of the difference between maximum force (kg) of right and left feet of Muscovy ducks (*Cairina moschata domestica*) as measured by a pressure sensitive walkway at serial time points following induction of lameness with intratarsal injection of monosodium urate solution. Asterisks represent a significant increase at hours 3 and 4.

**Fig 4:**
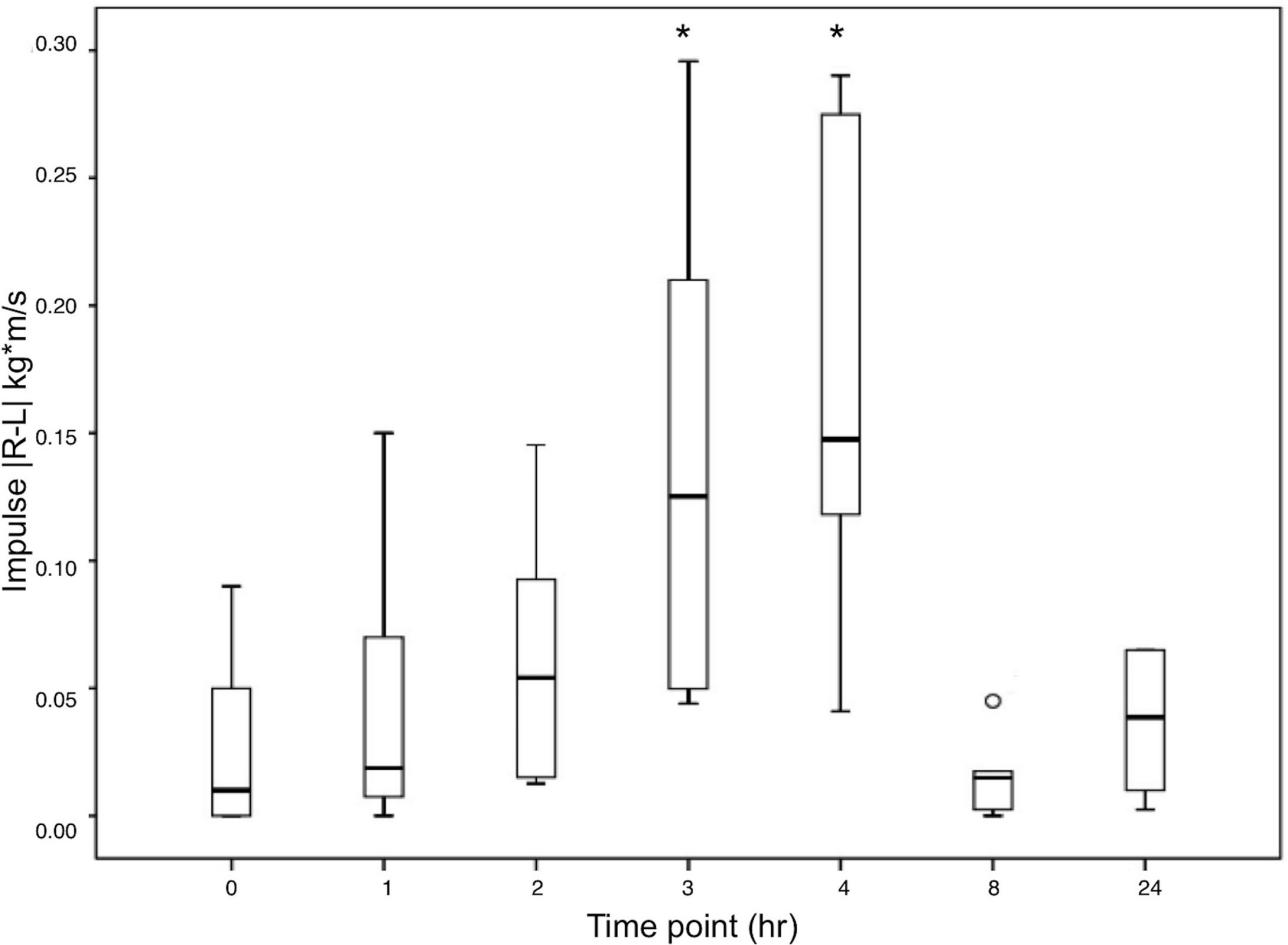
Impulse. Boxplot depicting the absolute value of the difference between impulse (kg*m/sec) of right and left feet of Muscovy ducks (*Cairina moschata domestica*) as measured by a pressure sensitive walkway at serial time points following induction of lameness with intratarsal injection of monosodium urate solution. Asterisks represent a significant increase at hours 3 and 4.

The six ducks included in experiment 2 tolerated anesthesia, MSU injection, recovery, and PSW trials well. No significant complications, such as bradycardia, tachycardia, cardiac arrest or apnea, were noted during anesthesia. Of the 144 trials analyzed in experiment 2, 12 were repeated once and 4 were repeated twice in order to obtain a complete set of 4 adequate trials. Ducks were anesthetized using isoflurane for an average of 7.2 min (range 5–13 min). Based on gross post-mortem examination, there were four males and two females. The repeated measures analysis of variance revealed that the |R-L| of maximum force in kilograms and in percentage of body weight (F_2.536,12.678_ = 23.619; p<0.0001), in addition to impulse in kilograms meter per second and in percentage of body weight meter per second (F_2.347,11,735_ = 14.845, p<0.0001), significantly differed at only the 3- and 4-hour time points in ducks with unilateral tarsal MSU injection (Figs 3 and 4). No other parameters significantly differed between baseline and post-MSU injection at any time point.

## Discussion

The PSW provided repeatable objective gait analysis in domestic ducks. All ducks successfully participated in the PSW protocol and no differences were identified in gait parameters between feet in normal ducks. Most ducks voluntarily walked along the PSW when placed at one end with a person standing behind them. In addition, the unilateral MSU arthritis induction method induced transient lameness in these ducks. This was the first study to the authors’ knowledge to combine MSU arthritis induction and PSW gait analysis to provide an avian nociception model with potential for analgesic efficacy studies in birds.

Most gait parameters had wCVs <0.25. The gait parameter with the highest wCV, or the lowest repeatability, was swing time (wCV = 0.46). The parameters that were found to be statistically significant in experiment 2, maximum force and impulse, had relatively low wCVs of 0.13 and 0.18 in experiment 1, respectively. It is possible that higher repeatability contributed to the ability of the PSW to detect significant differences between time points in these gait parameters.

Lameness was present during 3–4 hours post-MSU injection. However, there were resting periods between hours 4 and 8 and 8 and 24, and it is unknown at what point between hours 4 and 8, the lameness resolved. It is also unknown if the longer resting periods after the 4 hour time point may have had an effect on the ducks’ lack of lameness. While a transient method for lameness induction provides a more humane method than a permanent one, this result suggests that for an analgesic efficacy study, the lameness duration may not be sufficient to detect effects of medications for a clinically applicable time period. For example, tramadol plasma concentrations in this species maintained levels therapeutic for humans for at least 6 hours and the M1 metabolite maintained presumed therapeutic concentrations for at least 24 hours [30]. Future studies investigating a higher dose or concentration of MSU may yield a more effective lameness duration that would mimic true disease and allow for effects lasting as long as target medications. In addition, the type of mat covering the PSW should not be changed within a study because different cover mats can significantly affect the magnitude of gait parameters [32].

The gait parameters following MSU injection significantly reduced the maximum force (kg and % of body weight) and impulse (kg meter per second and % of body weight meter per second) put on the injected limb. There were no significant differences in step length between feet, indicating that the ducks were able to compensate and not change their stride length or shape. These findings differ from those found in other species of poultry with different lameness-causing diseases. High growth-rate breeding lines of broiler chickens (*Gallus gallus domesticus*) and Pekin ducks (*Anas platyrhynchos domesticus*), and birds fed an ad libitum diet, had a lower velocity, larger step width, and increased double support time (spent more time with two feet supporting the body) compared to low growth-rate lines and birds fed a restricted diet, respectively [1]. Additionally, turkeys kept on wet litter substrate developed pododermatitis, resulting in decreased velocity, increased double support time, decreased stride length, and increased stance time compared to those kept on dry litter [5,33]. Larger body size in chickens and ducks, and pododermatitis in turkeys affected different gait parameters compared with the MSU injections of the current study (maximum force and impulse). These findings indicate that different diseases (e.g. obesity, pododermatitis, osteoarthritis) cause different gait abnormalities.

Future studies warrant 1) testing a higher MSU dose to aim for longer lameness duration, 2) exploring objective gait analysis in other aquatic avian species using the PSW and 3) investigating efficacy studies for commonly used analgesics in waterfowl such as tramadol and meloxicam. Investigating the presence of inflammation in the joint after MSU injection via thermography, joint fluid analysis, and histopathology, and confirming the presence of MSU crystals as the cause of lameness by comparing to saline control injections were beyond the scope of this study but would be beneficial to evaluate in the future. Previous studies using MSU arthritis induction in birds also do not include a saline injection group likely due to restraints on animal numbers and earlier studies already having proven the efficacy of MSU compared to saline injections [11,12,13,14].

Species-specific gait assessment modalities are important due to the vast variability between gaits even among avian species. In addition, not only species-specific, but also disease-type specific pain models are needed to determine the most effective analgesic therapies for different avian species. In conclusion, objective gait analysis using the PSW offers a flexible technique to detect gait abnormalities in a variety of species with the potential to assess, monitor, and promote improvements in the welfare of birds with lameness causing diseases.
